# Sociodemographic and Lifestyle Characteristics Associated with Maternal Dietary Patterns in Mexico

**DOI:** 10.3390/nu16101451

**Published:** 2024-05-11

**Authors:** M. Karen Flores-García, María Luisa Pérez-Saldivar, Edgar Denova-Gutiérrez, Luis Rodolfo Rodríguez-Villalobos, Juan José Dosta-Herrera, Javier A. Mondragón-García, Alejandro Castañeda-Echevarría, M. Guadalupe López-Caballero, Sofía I. Martínez-Silva, Juan Rivera-González, Norma Angélica Hernández-Pineda, Jesús Flores-Botello, Jessica Arleet Pérez-Gómez, María Adriana Rodríguez-Vázquez, Delfino Torres-Valle, Jaime Ángel Olvera-Durán, Annel Martínez-Ríos, Luis R. García-Cortes, Carolina Almeida-Hernández, Janet Flores-Lujano, Juan Carlos Núñez-Enriquez, Vilma Carolina Bekker Mendez, Minerva Mata-Rocha, Haydeé Rosas-Vargas, David Aldebarán Duarte-Rodríguez, Silvia Jiménez-Morales, Juan Manuel Mejía-Aranguré, Lizbeth López-Carrillo

**Affiliations:** 1 Escuela de Salud Pública de México, Instituto Nacional de Salud Pública de México (INSP), Cuernavaca 62100, Morelos, Mexico; 2 Unidad de Investigación Médica en Epidemiología Clínica, Hospital de Pediatría, Centro Médico Nacional (CMN) “Siglo-XXI”, Instituto Mexicano del Seguro Social (IMSS), Mexico City 06720, Mexico; 3 Centro de Investigación en Nutrición y Salud, Instituto Nacional de Salud Pública de México (INSP), Mexico City 14080, Mexico; 4 Servicio de Pediatría, Hospital Pediátrico de Tacubaya, Secretaría de Salud de la Ciudad de México (SSCDMX), Mexico City 11870, Mexico; 5 Servicio de Cirugía Pediátrica, Hospital General “Gaudencio González Garza”, CMN “La Raza”, Instituto Mexicano del Seguro Social (IMSS), Mexico City 02990, Mexico; 6 Servicio de Cirugía Pediátrica, Hospital General Regional (HGR) No. 1 “Dr. Carlos Mac Gregor Sánchez Navarro”, Instituto Mexicano del Seguro Social (IMSS), Mexico City 03103, Mexico; 7 Servicio de Pediatría, Hospital General de Zona Regional (HGZR) No. 25, Instituto Mexicano del Seguro Social (IMSS), Mexico City 09100, Mexico; 8 Coordinación Clínica y Pediatría, Hospital Pediátrico de Coyoacán, Secretaría de Salud de la Ciudad de México (SSCDMX), Mexico City 04000, Mexico; 9 Hospital Pediátrico de Iztapalapa, Secretaría de Salud de la Ciudad de México (SSCDMX), Mexico City 09070, Mexico; 10 Hospital General Dr. “Gustavo Baz Prada”, Instituto de Salud del Estado de México (ISEM), Ciudad Nezahualcóyotl 57300, Estado de México, Mexico; 11 Coordinación Clínica y Pediatría del Hospital General de Zona 76, Instituto Mexicano del Seguro Social (IMSS), Ecatepec 55349, Estado de México, Mexico; 12 Coordinación Clínica y Pediatría, Hospital General “La Perla”, Instituto de Salud del Estado de México (ISEM), Ciudad Nezahualcóyotl 57820, Estado de México, Mexico; 13 Coordinación Clínica y Pediatría, HGR No. 72 “Dr. Vicente Santos Guajardo”, Instituto Mexicano del Seguro Social (IMSS), Tlalnepantla 54030, Estado de México, Mexico; 14 Coordinación Clínica y Pediatría del Hospital General de Zona 68, Instituto Mexicano del Seguro Social (IMSS), Ecatepec 55400, Estado de México, Mexico; 15 Coordinación Clínica y Pediatría del Hospital General de Zona 71, Instituto Mexicano del Seguro Social (IMSS), Chalco 56600, Estado de México, Mexico; 16 Servicio de Cirugía Pediátrica, HGR 1° Octubre, Instituto de Seguridad y Servicios Sociales de los Trabajadores del Estado (ISSSTE), Mexico City 07760, Mexico; 17 Hospital Regional “General Ignacio Zaragoza”, Instituto de Seguridad y Servicios Sociales de los Trabajadores del Estado (ISSSTE), Mexico City 09100, Mexico; 18 Delegación Regional Estado de México Oriente, Instituto Mexicano del Seguro Social (IMSS), Naucalpan 53370, Estado de México, Mexico; 19 Hospital General de Ecatepec “Las Américas”, Instituto de Salud del Estado de México (ISEM), Ecatepec 55076, Estado de México, Mexico; 20 Edificio Administrativo, UMAE, Hospital de Pediatría, Centro Médico Nacional (CMN) “Siglo-XXI”, Instituto Mexicano del Seguro Social (IMSS), Mexico City 06720, Mexico; 21 Jefatura de la División de Investigación en salud, UMAE, Hospital de Pediatría, Centro Médico Nacional (CMN) “Siglo-XXI”, Instituto Mexicano del Seguro Social (IMSS), Mexico City 06720, Mexico; 22 Unidad de Investigación Médica en Inmunología e Infectología, Hospital de Infectología “Dr. Daniel Méndez Hernández”, CMN “La Raza”, Instituto Mexicano del Seguro Social (IMSS), Mexico City 02990, Mexico; 23 Laboratorio de Biología Molecular de las Leucemias, Unidad de Investigación en Genética Humana, UMAE, Hospital de Pediatría, Centro Médico Nacional (CMN) “Siglo XXI”, Instituto Mexicano del Seguro Social (IMSS), Mexico City 06720, Mexico; 24 Laboratorio de Genética, Hospital de Pediatría, Centro Médico Nacional (CMN) “Siglo-XXI”, Instituto Mexicano del Seguro Social (IMSS), Mexico City 06720, Mexico; 25 Coordinación de Investigación en Salud, Anexo B, Unidad de Congresos, Centro Médico Nacional (CMN) “Siglo-XXI”, Instituto Mexicano del Seguro Social (IMSS), Mexico City 06720, Mexico; 26 Laboratorio de Innovación y Medicina de Precisión, Núcleo A, Instituto Nacional de Medicina Genómica (INMEGEN), Mexico City 14610, Mexico; 27 Genomica del Cáncer, Instituto Nacional de Medicina Genómica (INMEGEN), Mexico City 14610, Mexico; 28 Facultad de Medicina, Universidad Nacional Autónoma de México (UNAM), Mexico City 04360, Mexico; 29 Centro de Investigación en Salud Poblacional, Instituto Nacional de Salud Pública de México (INSP), Cuernavaca 62100, Morelos, Mexico

**Keywords:** dietary patterns, pregnant women, sociodemographic characteristics

## Abstract

There is scarce evidence on sociodemographic and lifestyle characteristics that may explain adherence to different dietary patterns (DPs) during pregnancy. Our aims were to identify dietary patterns in a sample of pregnant Mexican women and to describe their association with selected sociodemographic and lifestyle characteristics. This is a secondary cross-sectional analysis of 252 mothers of children that participated as controls in a hospital-based case–control study of childhood leukemia. We obtained parents’ information about selected sociodemographic characteristics, as well as alcohol and tobacco consumption. We also obtained dietary information during pregnancy. We identified DPs using cluster and factor analyses and we estimated their association with characteristics of interest. We identified two DPs using cluster analysis, which we called “Prudent” and “Non healthy”, as well as three DPs through factor analysis, namely “Prudent”, “Processed foods and fish”, and “Chicken and vegetables”. Characteristics associated with greater adherence to “Prudent” patterns were maternal education, older paternal age, not smoking, and being a government employee and/or uncovered population. Likewise, the “Processed foods and fish” pattern was associated with greater maternal and paternal education, as well as those with less household overcrowding. We did not identify sociodemographic variables related to the “Chicken and Vegetables” pattern. Our results may be useful to identify target populations that may benefit from interventions aimed to improve individual dietary decisions during pregnancy.

## 1. Introduction

During pregnancy, many women do not meet the requirements for the consumption of vegetables, cereals, folates, iron, and calcium [1]. The lack of adherence could be as a result of these recommendations, such as the dietary guidelines of the World Health Organization, focusing on food groups and/or individual nutrients that, one-by-one, are a difficult requirement to fully meet [1,2]. Since foods are not consumed in isolation, DPs have been proposed, which reflect dietary consumption as a whole [3]. These patterns that include combinations of foods or food groups are commonly adopted across different populations [3,4]. “A priori” DPs have been established, for instance, the “Mediterranean” DP, that includes fruits, vegetables, legumes, oilseeds, and fish [5]. In addition, “A posteriori” patterns have also been described and are obtained from reported information on food consumption in specific populations, for example, the “Prudent” and the “Western” [6]. These patterns are not found homogeneously across countries, possibly due to cultural differences and accessibility to local foods [7]. However, in several countries, dietary patterns have been similarly defined by the inclusion of common food groups, such as “Western” (white bread, red and processed meats, and high-fat dairy products) and “Prudent” (fruits, vegetables, whole grains, and fish) [6].

Furthermore, attachment to specific patterns depends on differences in sociodemographic and lifestyle characteristics [8]. It has been reported that older people generally adhere to healthier patterns [9]. Additionally, pregnant women [9], with a higher educational [10,11,12] and socioeconomic level [12,13], who perform physical activity [12,13,14], show a greater attachment to healthy patterns, in contrast to smokers [13,14]. Dietary reference guidelines should be based on DP adherence, considering the main sociodemographic drivers [3,4]; however, this is not a prevalent strategy in many countries in Latin America

In Mexico, some dietary patterns have been identified in pregnant women. The “Rural” and “Traditional” patterns [15] that comprise many corn-based local foods have not been observed in other populations that consume mainly wheat [6]; the “Healthier” [16] DP is like the “Prudent” pattern, since it contains fruits and vegetables; and the “Refined food” pattern [15] is like the “Western” pattern that includes red meat and processed foods [6]. In addition, in this country, there is a “Mixed” pattern that combines processed foods; sugar-sweetened beverages that include local traditional beverages, usually prepared with water, fruit, and table sugar; as well as the cereal group including “tortilla” that is the most popular corn-based food in Mexican culture that might reflect the transition to the Western diet [16]. 

This report aims to identify dietary patterns and describe their association with selected sociodemographic and lifestyle characteristics in a sample of pregnant women from the Mexico City Metropolitan Area.

## 2. Materials and Methods

### 2.1. Materials, Study Design, and Participants

We developed a cross-sectional secondary analysis using data from 252 women aged 14 to 46 years, who participated as controls in the original hospital-based case–control study about child leukemia that was carried out during the period from 2010 to 2019 in the Mexico City Metropolitan Area [17]. A detailed description of the original study is described elsewhere [18].

### 2.2. Interviews

#### 2.2.1. Parents Interviews

Both parents of the participating children were interviewed directly, in their respective hospitals, by previously trained personnel, regarding their sociodemographic characteristics and tobacco consumption. Mothers only were also interviewed about diet and alcohol consumption during pregnancy.

#### 2.2.2. Maternal Diet

Mothers were interviewed about the consumption of 109 foods and beverages and seven dishes during their pregnancy with the participating child, through a food frequency questionnaire (FFQ). The reproducibility of this instrument was evaluated in Mexican women, to whom the questionnaire was applied twice, at an interval of one year, while its validity was estimated using 24 h recalls at 3-month intervals as a reference. Details of this validation have been previously published [19].

According to the methodology suggested by Willett et al. [20], the food questionnaire included 10 response options for the frequency of consumption, ranging from “never” to “6 or more times a day”, as well as predetermined portions for each food, as follows: a glass (e.g., milk and wine), a cup (e.g., yogurt, some fruits and vegetables, tea, juices, and alcoholic and non-alcoholic beverages), a spoon (for oils, sour cream, sauces, and nuts), a slice (for cheeses, some fruits, and meats), a plate (e.g., legumes and local dishes), and a piece (for some fruits and breads).

Total energy intake was estimated by adding up the kilocalorie daily intake of foods and dishes. The nutritional and energy content was calculated using composition tables of the United States Department of Agriculture [21,22] that include a wide variety of foods; for the local foods not included, such as tejocote, we used, as nutritional reference content, the tables of the Salvador Zubirán National Institute of Medical Sciences and Nutrition [23]. Two foods (soy juice and soy beer) with a very low prevalence of consumption (5.8 y 0.28%, respectively) were not found in those tables, so they were not included in the total energy intake calculation. Since some fruits and vegetables are only consumed at certain times of the year, their energy intake was adjusted according to the time of their availability in the market, for example, only 50% of the kilocalories of plums were considered, as they are only available for 6 months of the year [24]. A consumption of less than 525 kcal/day was established as a criterion for implausible values of total energy intake; however, all women met this criterion.

#### 2.2.3. Food Groups

Foods and beverages contained in the FFQ were categorized into 30 food groups, considering the similarity in the content of macro and micronutrients (e.g., fat, carbohydrates, protein, vitamins, and sodium), the content of added sugar (added or not), type of fat (saturated or vegetable), etc. Some of the individual foods were considered as groups by themselves, because their nutritional content did not meet the criteria to belong to a particular group (e.g., eggs, chicken, blueberries, and avocado, among others) or because of their high consumption among the population (e.g., corn tortilla and corn) (Table 1). 

#### 2.2.4. Dietary Patterns

Dietary patterns are defined as the amounts, proportions, variety, and combination of different food groups, foods, beverages, and nutrients, as well as the frequency with which they are typically consumed [3]. For the a posteriori estimation of DPs, the energy intake of each food group was converted to a percentage of the total daily energy intake and was standardized with a Z score, to further obtain dietary patterns through cluster and factor analysis. The first was carried out using the K-means methodology, which allows assigning cases to a fixed number of mutually exclusive clusters (patterns). To stabilize the accuracy of the results obtained using this technique, the algorithm was run at least 100 times, as other authors have suggested [25]. On the other hand, through a factor analysis, we obtained the loading factors for each food group. These factors were orthogonally rotated to maximize their differences. The dietary patterns were defined according to the minimum number of different food groups (n = 7) that are consumed per day in other populations and that have been reported elsewhere (3–9) [26], with an absolute load factor greater than or equal to 0.25 and an eigenvalue greater than 1.5. Unlike the cluster analysis, in factor analysis, all women have an attachment score to each possible existing pattern [27]. We named DPs according to the food groups that predominated in each one of them.

### 2.3. Statistical Analysis

We used t-Student, Mann–Whitney U, Kruskal–Wallis, ANOVA, or X2 tests to compare medians, means, or percentages, respectively, of sociodemographic and lifestyle characteristics of the child’s mother and father, according to the patterns obtained, using both cluster analysis and the adherence tertiles (high, medium, or low) of the factor analysis pattern scores. The variables smoking (before and during pregnancy), consumption of alcohol, iron, minerals, and vitamins were classified as yes and no. The health institution was divided into two categories—SS or ISSSTE, and IMSS. The education of both the father and the mother was classified into three categories according to years of study, as follows: ≤9, >9–≤12, and >12. The level of overcrowding was calculated using the person/bedroom ratio and was categorized into ≤3 and >3; this variable was used as a proxy for socioeconomic status.

Variables that differed in any of the comparisons of the extreme categories of the patterns both using cluster and using factor analysis (paternal education, overcrowding, health institution, maternal education, and smoking before pregnancy) were maintained in the multivariate models.

Through logistic regression models, we estimated the odds ratios and their corresponding confidence intervals for each sociodemographic variable, as well as the “Prudent” vs. “Non healthy” pattern that resulted from cluster analysis. In addition, a linear trend was estimated using the following variables as continuous: overcrowding, maternal education, paternal age at pregnancy, and paternal education. We further used linear regression models to determine associations between the score for each dietary pattern obtained using factor analysis and the selected sociodemographic and lifestyle variables. All models were adjusted for the variables suggested by the software.

A value of *p* < 0.05 was considered statistically significant. All statistical analyses were performed with the statistical package Stata 14.0.

## 3. Results

Different dietary patterns were identified using both methodologies. In the cluster analysis, we found two patterns that we called “Prudent” and “Non healthy”; while in the factorial analysis, we identified three—“Prudent”, “Processed foods and fish”, and “Chicken and vegetables”. 

Food groups that provided the highest percentage of energy were considered to characterize the patterns obtained using cluster analysis. The “Prudent” pattern contained cereals, legumes, non-citrus fruits, citrus fruits, *atole*, and fish and shellfish, among others; in contrast, the “Non healthy” pattern was mainly characterized by the consumption of corn tortilla, red and processed meats, vegetable fats, high-fat dairy products with added sugar, and eggs.

On the other hand, patterns that were obtained through factor analysis were characterized by extreme load factors of each food group—the “Prudent” pattern was mainly characterized by a high intake of non-citrus fruits, cruciferous and green leafy vegetables, root vegetables, legumes, and corn, as well as a low intake of soft drinks, coffee and tea, and dairy products with added sugar; the “Processed foods and fish” pattern was mainly represented by a high intake of high-fat dairy products, dairy products with added sugar, cereals high in fat and sugar, and fish and shellfish, among others, as well as a low consumption of corn tortillas. Lastly, the “Chicken and Vegetables” pattern included a high contribution of allium vegetables, other vegetables, vegetable oils, chicken, and coffee and tea. The variance explained in each pattern was 8.7%, 7.7%, and 7.4%, respectively (Table 1). 

Compared with women who adhered mostly to the “Prudent” pattern, obtained using cluster methodology, the women who adhered to the “Non healthy” pattern had fewer years of study, smoked more before pregnancy, and had a partner of a younger age. Likewise, comparing the categories of high and low adherence in each of the three patterns identified using factorial analysis, women in the highest tertile of consumption of the “Prudent” pattern smoked less before and during pregnancy, consumed more supplements of minerals and iron during pregnancy, had an older partner, and most of them belonged to the Secretary of Health or the Institute of Security and Social Services for State workers. In contrast, women in the highest tertile of the “Processed foods and fish” pattern smoked more before pregnancy, had more years of study, and had a partner with a higher level of education; in addition, they had a lower person/bedroom ratio. No differences were found when comparing the extreme categories of adherence within the “Chicken and vegetables” pattern (Table 2).

Compared to the “Non healthy” pattern, women with a higher level of education had a greater adherence to the “Prudent” pattern (OR = 4.34; 95% CI: 1.70, 11.09) (*p*-trend = 0.004). In contrast, women entitled to IMSS (OR = 0.33, 95% CI: 0.16, 0.69) and those who smoked before pregnancy (OR = 0.42, 95% CI: 0.21, 0.82) were less likely to adopt this pattern (Table 3). 

Regarding the dietary patterns obtained using factor analysis, women entitled to IMSS (β = −0.46; 95% CI −0.74, −0.17) and those who partook in smoking before pregnancy (β= −0.47; 95% CI −0.74, −0.21) showed less adherence to the “Prudent” pattern. In addition, the “Processed foods and fish” pattern was related to a higher level of the mother’s education (β= 0.97; 95% CI 0.59, 1.36) (*p*-trend = <0.001), less overcrowding (β = −0.21; 95% CI −0.45, 0.04) (*p*-trend = 0.046), and being entitled to IMSS (β = −0.29; 95% CI −0.57, −0.02). In contrast, the “Chicken and vegetables” pattern resulted in a suggestive association with a higher person/bedroom ratio (β = −0.23; 95% CI −0.03, 0.50) (*p*-trend = 0.018) (Table 4).

## 4. Discussion

In this sample of pregnant Mexican women, two dietary patterns were identified, through cluster analysis, which we named “Prudent” and “Unhealthy”, as well as three patterns identified using factor analysis—“Prudent”, “Processed foods and fish”, and “Chicken and vegetables”. In the multivariate models, the characteristics associated with a greater attachment to “Prudent” patterns” were a higher maternal education, non-smoking, and being entitled to the SS or the ISSSTE. Likewise, the pattern “Processed foods and fish” was associated with a higher maternal education and with less overcrowding. No sociodemographic variables were identified with the “Chicken and vegetables” pattern.

According to our results, higher education was associated with both the “Prudent” pattern, considered healthy, and the “Processed foods and fish” pattern, considered unhealthy. Some authors have described that, in Mexico, a higher educational level is related to the adoption of a healthy lifestyle, including a healthy diet [28,29,30]; however, it has also been observed that people with higher education might have a higher socioeconomic level and possibilities of acquiring more expensive products such as fish and shellfish, as well as ultra-processed products [28,31,32]. For example, education was positively associated with higher quality and diversity of diet, as well as adherence to healthy dietary patterns, in studies conducted in countries of medium income such as Mexico [33]. However, it has also been seen that higher education is not always associated with a high-quality diet, when knowledge in nutrition is lacking [31]. In addition, other studies have suggested that higher education was positively associated with unhealthy patterns and a higher consumption of ultra-processed products [28,34]. The above evidence agrees with our findings.

In this report, we did not have a direct measurement of socioeconomic level, but we used the person/bedroom ratio as a proxy, which has been inversely associated with both socioeconomic [35] and educational level [36]. After adjusting for sociodemographic characteristics, we found that a greater person/bedroom ratio was associated with a lower adherence to the pattern “Processed foods and fish”, possibly due to what has been expressed in the previous paragraph in relation to the increased purchasing power related to education and socioeconomic level. In addition, it is possible that the nutritional transition from traditional diets to more expensive Westernized diets, which has been identified in middle-income countries such as Mexico, produces a slip towards the consumption of these foods among higher socioeconomic levels [37].

These findings are consistent with those previously observed. In a study performed in Mexico, it was reported that people with high educational and socioeconomic levels had a higher consumption of ultra-processed foods [34]. Moreover, according to a systematic review that included 45 studies from countries of middle income, people with a higher socioeconomic level consumed more fats and processed foods [28]. Maternal smoking was negatively associated with “Prudent” patterns, which is consistent with previous studies [10,13,14,38]; although paternal smoking has not been independently associated with adherence to any dietary pattern, it has been observed that partners who smoke adhere less to healthy patterns [39,40]. In this study, we observed that most female partners of male smokers were also smokers (68%).

In addition, we observed that mothers entitled to the IMSS were less attached to the “Prudent” pattern and, although we do not have a clear explanation, it might be due to the smaller amount of available information on prevention, compared to other health institutions.

To interpret our results, some methodological considerations must be taken into account. The study sample was selected only from public hospitals, so it might not represent the diet of pregnant women in private hospitals. The total median energy consumption in the studied women (2191 Kcal/day) was similar to that reported by other authors, for pregnant women from Mexico City treated in primary-level federal hospitals (2166 Kcal/day), where higher socioeconomic levels are not represented [41]. As such, we think that our population is not likely to be biased in their selection. Furthermore, a limitation of our study is the lack of information on the health status of the participating women. According to some reports, between 13 and 29.6% of pregnant women may have gestational diabetes in Mexico [42]; it is possible that some participants have modified their eating patterns due to this or other situations during pregnancy. However, we cannot estimate in what direction these conditions could have affected our results, since, among other limitations, we do not have information on the pre-pregnancy body mass index and/or maternal gestational weight gain. In addition, the non-differential measurement error, inherent in the use of dietary questionnaires, is a limitation that might attenuate the associations reported in this study. Also, despite the fact that we found some significant results, we cannot rule out the possibility that some associations may have been lost, due to a lack of power. On the other hand, due to the dietary differences under the diverse sociocultural context worldwide, the dissimilarities in periods of pregnancy in which the diet is evaluated, the heterogeneity in the conformation of the food groups that further define the dietary patterns, and the comparison of dietary patterns throughout the countries is constrained [16]. However, the identification of a “Prudent” or “Healthy” [6] pattern is consistent in most studies, as occurred in this report.

To our knowledge, this is the first study that evaluates some of the sociodemographic characteristics that are associated with adherence to specific dietary patterns in pregnant Hispanic women, using two methodologies. Further research is warranted to consider other factors that may be related to the adherence of DPs at different stages of pregnancy such as lifestyle, culture, traditions, religion, and social support in Latin American countries.

## 5. Conclusions

In conclusion, maternal education, health institution, and smoking are associated factors with the adherence to different DPs. This information may be useful to develop interventions in target populations, to improve dietary decisions during pregnancy.

## Figures and Tables

**Table 1:** Food groups, items, and maternal dietary patterns using cluster or factor analysis.

		Dietary Pattern						
Foods or Food Groups	Food Items	Cluster				Factor Analysis		
		Prudent		Non Healthy		Prudent ^e^	Processed Foods and Fish ^f^	Chicken and Vegetables ^g^
		% Energy/Day				Factor Loading		
		Mean	SD	Mean	SD			
High fat dairy products	Milk, Oaxaca cheese, fresh cheese, Manchego cheese	5.04	2.89	**5.38**	5.35	--	0.28	--
Dairy with added sugar	Ice cream, yogurt	3.55	3.09	**3.72**	3.99	−0.28	0.33	--
Citrus fruits	Orange, orange juice, tangerine, tangerine juice, grapefruit, grapefruit juice	**4.01**	2.64	2.88	2.62	--	0.34	--
Non-citrus fruits	Banana, peach, apple, grapes, strawberries, melon, watermelon, mango, pear, cactus fruit, papaya, pineapple, plum, blackberry, mamey, zapote ^a^	**9.49**	4.27	5.56	3.13	0.30	0.44	--
Dehydrated cranberries	Dehydrated cranberries	**1.03**	2.07	0.17	0.48	--	0.46	--
Eggs	Eggs	3.03	2.34	**4.19**	4.09	--	--	--
Chicken	Chicken	1.22	0.74	**1.28**	0.81	--	0.34	0.28
Processed meats	Sausage, ham, chorizo, bacon	2.55	2.36	**2.80**	2.20	--	--	--
Red meat	Beef, pork, barbecue, carnitas, liver, cecina ^b^	5.64	4.32	**6.16**	4.35	--	--	--
Fish and shellfish	Tuna, shellfish, sardine, fish	**2.29**	2.96	1.16	1.04	--	0.45	--
Foods high in saturated fats	Pork rinds, sour cream, butter, mayonnaise, lard	1.89	1.11	**2.12**	1.26	--	--	--
Cruciferous vegetables	Broccoli, cauliflower, cabbage	**0.78**	0.57	0.28	0.34	0.59	--	--
Allium vegetables	Onion, garlic	0.27	0.14	**0.34**	0.19	--	--	0.83
Green leafy vegetables	Purslane, spinach, lettuce, parsley	**0.30**	0.17	0.10	0.07	0.76	--	--
Other vegetables	Zucchini, stewed tomato, raw tomato, chili, squash flower, chayote, nopal ^c^	5.40	5.4	**5.74**	2.77	--	--	0.85
Corn	Corn	**1.47**	1.48	1.01	1.08	0.35	--	--
Root vegetables	Potato, carrot, beetroot	**3.28**	1.84	1.87	1.32	0.50	--	--
Soy products	Soy-based drink (soy milk), tofu (soy cheese), textured soy protein (soy meat)	**0.20**	0.58	0.05	0.26	0.26	--	--
Legumes	Lentils, beans, broad beans, peas	**6.8**	3.63	5.78	4.35	0.41	--	--
Soy sauce	Soy sauce	**0.00**	0.01	**0.00**	0.00	--	0.36	--
Canned chili peppers	Canned chili peppers	0.03	0.04	0.03	0.05	--	--	--
Corn tortilla	Corn tortilla	10.93	6.9	18.99	12.73	--	−0.79	--
Cereals	Flour tortilla, white bread, rice, pasta, oatmeal, breakfast cereal, bolillo	**15.47**	5.53	12.85	4.91	--	--	--
Cereals high in fat and sugar	Cookies, cake, sweet bread	3.33	2.14	**3.98**	3.04	−0.30	0.30	--
Soft and energy drinks	Soft drink, energy drink	1.52	1.48	**3.16**	3.64	−0.33	--	--
Alcoholic drinks	Red wine, white wine, beer, liquors/spirits (rum, brandy, tequila)	0.16	0.33	**0.20**	0.39	--	--	--
Coffee and tea	Coffee, herbal tea, green tea, black tea	1.04	0.96	**1.99**	1.47	−0.38	--	0.30
Atole	Atole ^d^	**2.55**	2.16	1.29	1.66	0.47	--	--
Avocado	Avocado	**1.7**	1.32	1.18	1.32	0.25	--	--
Vegetable fats	Vegetable oil, margarine, corn oil, olive oil, soy oil	5.02	2.8	**5.74**	3.60	--	--	0.37

^a^ local fruit, ^b^ local salted beef meat, ^c^ local cactus leaf, ^d^ local corn-based drink, ^e^ explained variance 8.72%, ^f^ explained variance 7.67%, ^g^ explained variance 7.39%. Bold numbers are the highest in the corresponding pattern.

**Table 2:** General characteristics of the study sample according to maternal dietary patterns.

	Cluster Analysis			Factor Analysis								
	Prudent	Non Healthy		Prudent			Processed Foods and Fish			Chicken and Vegetables		
			*p*-Value	Tertile 1	Tertile 3	*p*-Value	Tertile 1	Tertile 3	*p*-Value	Tertile 1	Tertile 3	*p*-Value
Mother												
Age at pregnancy, years [mean(SD)]	25.76 (6.76)	25.50 (6.15)	0.333	25.74 (6.66)	25.83 (6.49)	0.468	25.76 (6.89)	25.84 (6.19)	0.357	25.06 (6.65)	26.29 (6.60)	0.295
Education, years [p50 (p10, p90)]	11.00 (8.00, 15.00)	9.00 (6.00, 13.00)	0.031	11.00 (6.00, 13.60)	10.00 (7.00, 14.00)	0.705	9.00 (6.00, 12.00)	12.00 (9.00, 15.60)	<0.001	11.00 (6.00, 13.00)	10.30 (6.00, 14.00)	0.581
Smoking before pregnancy, yes %	20.00	35.56	0.018	45.24	19.05	0.001	22.62	39.29	0.063	38.10	28.57	0.163
Smoking during pregnancy, yes %	1.33	3.39	0.364	5.95	0.00	0.061	2.38	4.76	0.358	3.57	1.19	0.556
Alcohol consumption during pregnancy, yes %	6.67	14.69	0.076	16.67	9.52	0.320	10.71	14.29	0.773	16.67	8.33	0.256
Iron consumption during pregnancy, yes %	88.00	84.75	0.500	78.57	91.67	0.049	83.33	85.71	0.506	80.95	90.48	0.211
Minerals consumption during pregnancy, yes %	6.67	6.21	0.893	1.19	8.33	0.057	8.33	7.14	0.420	7.14	2.38	0.154
Vitamins consumption during pregnancy, yes %	94.67	94.35	0.920	94.05	94.05	0.927	91.67	95.24	0.374	92.86	92.86	0.298
State of residence, %												
Mexico City	60.00	49.15	0.120	52.38	58.33	0.300	47.62	60.71	0.170	58.33	47.42	0.370
Mexico State	40.00	50.85		47.62	41.67		52.38	39.29		41.67	52.38	
Health institution, %												
SS and ISSSTE	72.00	62.15	0.130	57.14	75.00	0.050	63.10	60.71	0.310	63.10	61.90	0.470
IMSS	28.00	37.85		42.86	25.00		36.90	39.29		36.90	38.10	
Person/room ratio, [p50(p10, p90)]	3.00 (1.50, 5.50)	3.00 (1.70, 6.00)	0.121	3.00 (1.70, 5.00)	3.00 (1.70, 6.00)	0.558	3.50 (1.75, 6.00)	2.40 (1.50, 5.00)	<0.001	3.00 (1.50, 5.00)	3.00 (1.70, 6.00)	0.877
Father												
Age at pregnancy, years [mean(SD)]	29.84 (8.58)	28.44 (6.83)	0.018	28.53 (7.28)	29.73 (8.44)	0.036	28.81 (7.65)	28.66 (7.02)	0.700	28.88 (8.36)	28.80 (6.93)	0.131
Education, years [p50 (p10, p90)]	9.00 (6.00, 16.00)	9.00 (6.00, 13.00)	0.777	10.00 (7.00, 14.00)	9.00 (6.00, 15.50)	0.468	9.00 (6.00, 12.00)	11.00 (8.00, 15.00)	<0.001	10.00 (6.00, 15.00)	9.00 (6.00, 14.00)	0.488
Smoking before pregnancy, yes %	53.52	59.88	0.361	65.85	49.37	0.105	54.43	57.32	0.600	53.75	56.10	0.369

SS, Secretary of Health; ISSSTE, Institute of Security and Social Services for State workers; IMSS, Mexican Institute of Social Security.

**Table 3:** Dietary patterns associated with selected characteristics.

		Cluster Analysis
		Prudent vs. Non Healthy
Variable	(n)	OR (95% CI) *
Mother		
Person/room ratio		
≤3	95/46	1.00
>3	82/29	0.81 (0.44, 1.48)
*p*-trend		0.388
Education		
≤9	91/29	1.00
>9–≤12	65/29	1.77 (0.91, 3.45)
>12	21/17	4.34 (1.70, 11.09)
*p*-trend		0.004
Health institution		
SS and ISSSTE	54/110	1.00
IMSS	67/21	0.33 (0.16, 0.69)
Smoking before pregnancy		
No	115/60	1.00
Yes	62/15	0.42 (0.21, 0.82)
Smoking during pregnancy		
No	121/124	1.00
Yes	4/3	1.10 (0.11, 10.57)
Father		
Age at pregnancy		
≥31	66/32	1.00
<31–>24	50/19	0.76 (0.37, 1.56)
≤24	61/24	0.75 (0.38, 1.48)
*p*-trend		0.094
Education		
≤9	94/39	1.00
>9–≤12	54/19	0.78 (0.39, 1.57)
>12	29/17	1.16 (0.49, 2.75)
*p*-trend		0.431
Smoking before pregnancy		
No	49/53	1.00
Yes	75/66	0.87 (0.48, 1.58)

SS, Secretary of Health; ISSSTE, Institute of Security and Social Services for State workers; IMSS, Mexican Institute of Social Security. * Adjusted by fathers’ education, overcrowding, health institution, mothers’ education, and smoking before pregnancy.

**Table 4:** Sociodemographic and lifestyle determinants of dietary pattern scores during pregnancy.

		Factor Analysis					
		Prudent Pattern		Processed Foods and Fish		Chicken and Vegetables	
Variable	(n)	β	(95% CI)	β	(95% CI)	β	(95% CI)
Mother							
Person/room ratio							
≤3	141	Ref.		Ref.		Ref.	
>3	111	0.15	(−0.11, 0.40)	−0.21	(−0.45, −0.04)	0.23	(−0.03, 0.50)
*p*-trend		0.291		0.046		0.018	
Education							
≤9	120	Ref.		Ref.		Ref.	
>9–≤12	94	0.12	(−0.16, 0.40)	0.54	(0.27, 0.81)	−0.12	(−0.41, 0.17)
>12	38	0.00	(−0.40, 0.40)	0.97	(0.59, 1.36)	0.11	(−0.30, 0.53)
*p*-trend		0.186		<0.001		0.204	
Health institution							
SS and ISSSTE	164	Ref.		Ref.		Ref.	
IMSS	88	−0.46	(−0.74, −0.17)	−0.29	(−0.57, −0.02)	−0.12	(−0.41, 0.17)
Smoking before pregnancy							
No	175	Ref.		Ref.		Ref.	
Yes	77	−0.47	(−0.74, −0.21)	0.24	(−0.01, 0.49)	−0.25	(−0.52, 0.03)
Smoking during pregnancy							
No	245	Ref.		Ref.		Ref.	
Yes	7	−0.34	(−1.1, 0.23)	0.71	(−0.03, 1.45)	−0.15	(−0.95, 0.64)
Father							
Age at pregnancy							
≥31	98	Ref.		Ref.		Ref.	
>24–<31	69	0.00	(−0.30, 0.30)	0.01	(−0.29, 0.30)	−0.13	(−0.44, 0.17)
≤24	85	−0.13	(−0.42, 0.15)	−0.18	(−0.45, 0.10)	−0.04	(−0.34, 0.26)
*p*-trend		0.169		0.144		0.366	
Education							
≤9	133	Ref.		Ref.		Ref.	
>9–≤12	73	−0.14	(−0.43, 0.14)	0.07	(−0.21, 0.34)	0.01	(−0.28, 0.31)
>12	46	0.16	(−0.21, 0.53)	0.10	(−0.26, 0.46)	−0.13	(−0.51, 0.26)
*p*-trend		0.156		0.525		0.934	
Smoking before pregnancy							
No	102	Ref.		Ref.		Ref.	
Yes	141	−0.21	(−0.46, 0.04)	−0.08	(−0.32, 0.16)	0.12	(−0.14, 0.38)

SS, Secretary of Health; ISSSTE, Institute of Security and Social Services for State workers; IMSS, Mexican Institute of Social Security. * Adjusted by father’s education, overcrowding, health institution, mothers’ education, and smoking before pregnancy.

## Data Availability

The data presented in this study are available on request from the corresponding author due to ethical reasons.
